# Watching Genes Loop the Loop

**DOI:** 10.1371/journal.pbio.1001592

**Published:** 2013-06-18

**Authors:** Roland G. Roberts

**Affiliations:** Public Library of Science, Cambridge, United Kingdom

Most cells of a given species contain essentially the same complement of genes, yet individual genetically identical cells can assume dramatically different appearances and behaviours. This is achieved largely by the regulation of the transcription of individual genes, orchestrated by the specific binding of proteins to nearby sites in the genome—a principle first recognised 50 years ago by François Jacob (who just died this April) and Jacques Monod from their work on the paradigmatic *lac* operon in *Escherichia coli*.

François Jacob went on to establish the second textbook workhorse of transcriptional regulation—the lambda repressor system. Bacteriophage lambda is a virus that infects *E. coli* but then makes a two-way decision to either take a nap in the host genome or to “go viral” and burst the cell, releasing a hundred or so bacteriophage babies (lysogeny versus lysis, in the parlance). The dormant lysogenic phase can be perpetuated stably for many bacterial generations, and is maintained by the binding of the lambda repressor to the phage genome (now lodged within the host bacterial genome).

But this is where it emerged that genes aren't only regulated by the binding of proteins to sites in their immediate vicinity, as is the case of the simple *lac* operon. Instead, in many cases, sites a considerable distance away can also influence the decision whether to transcribe or not to transcribe a gene. The lambda repressor molecules in fact bind to two separate regions in the phage genome that are 2.3 kilobases apart (about 700 nm on a straight DNA molecule, about 200 nm on average in solution, and substantially less inside a real live bacterium). Direct interactions between the two clusters of lambda repressors are then assumed to cause looping of the intervening DNA, bringing the two previously distant sites together. This shuts down almost all phage transcription but enhances transcription of the repressor gene, which lies next to one of the two sites, thereby making more repressor protein and perpetuating the dormant state. Many bacterial genes and most eukaryotic genes are thought to be regulated in ways that involve DNA looping.

The problem has been that this looping process, while widely assumed to be true, has been inferred from clever but indirect experiments, often *in vitro*, and usually by measuring the average behaviour of many thousands of molecules. The authors of a new study, just published in *PLOS Biology*, however, have gone one better. Zach Hensel, Jie Xiao, and colleagues, at Johns Hopkins University School of Medicine, have used some nifty technology to visualise the lambda repressor looping process in real-time and in single, intact, living bacterial cells. To achieve this impressive feat, they modified the bacteriophage DNA so that sequences flanking one end of the loop would bind a red fluorescent protein (LacI-mCherry), while sequences flanking the far end would bind a green one (TetR-EYFP). Measuring the distance between the resulting red and green spots would then in principle allow them to monitor the looping process (see the Figure).

**Figure:**
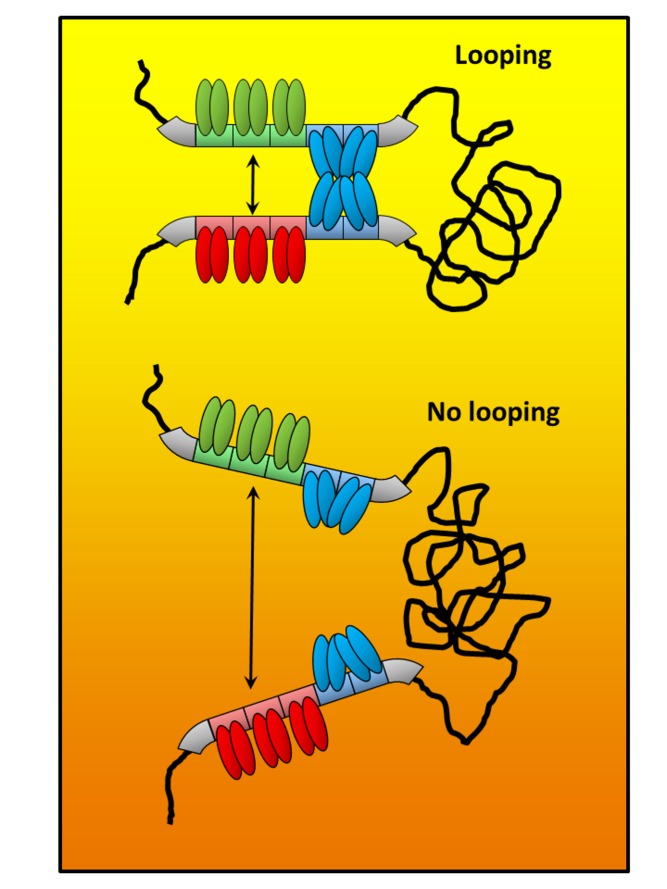
Red LacI-mCherry and green TetR-EYFP allowed the authors to directly visualise DNA looping caused by eight lambda repressor molecules (blue) in individual living bacteria by measuring the distance between red and green spots (double-headed arrows).

There are some severe complications, of course: because of the stochastic nature of single-molecule interactions, not all fluorescent protein molecules are fluorescent, not all repressor sites are bound by repressors, and not all repressors interact with each other. To make things worse, DNA in a bacterial cell is already highly compacted, so the two ends of the loop will be quite close together even in the absence of looping. This called for some serious single-molecule, super-resolution imaging and statistical modelling to allow bona fide looping behaviour to emerge from the nonspecific contacts between different regions of the genome (imagine trying to spot specific looping behaviour from chance encounters inside a ball of wool).

Despite these obstacles, the authors were able to show that lambda repressor did indeed cause looping, and were able to correlate this with transcription. By running the experiment with different versions of the lambda genomic DNA sequence and with mutated version of the repressor, they could show that the looping was dependant on the ability of the repressor to bind specific DNA sequences, and its ability to form the octameric complexes assumed to mediate such looping.

It seems fitting that four decades after Jacob's initial work we are finally moving into the realm of directly observing phenomena that had to be so cleverly inferred from macroscopic read-outs. Xiao and colleagues' technical advance, having cut its teeth on this well-known model system, is now ready to examine the behaviour of genomes in numerous less familiar scenarios where looping is suspected, both in prokaryotes and eukaryotes. Lights, camera, action!

**Hensel Z, Weng X, Lagda AC, Xiao J (2013) Transcription-Factor-Mediated DNA Looping Probed by High-Resolution, Single-Molecule Imaging in Live** ***E. coli*** **cells. doi:10.1371/journal.pbio.1001591**

